# Small head movements increase and colour noise in data from five video-based P–CR eye trackers

**DOI:** 10.3758/s13428-021-01648-9

**Published:** 2021-08-06

**Authors:** Kenneth Holmqvist, Saga Lee Örbom, Raimondas Zemblys

**Affiliations:** 1 Institute of Psychology, Nicolaus Copernicus University in Torun, Torun, Poland; 2 Department of Psychology, Regensburg University, Regensburg, Germany; 3 Department of Computer Science and Informatics, University of the Free State, Bloemfontein, South Africa; 4 Gothenburg, Sweden

**Keywords:** Eye tracker, Data quality, Head movements, Precision, Oculomotor drift, Corneal reflection, Pupil size artefact

## Abstract

We empirically investigate the role of small, almost imperceptible balance and breathing movements of the head on the level and colour of noise in data from five commercial video-based P-CR eye trackers. By comparing noise from recordings with completely static artificial eyes to noise from recordings where the artificial eyes are worn by humans, we show that very small head movements increase levels and colouring of the noise in data recorded from all five eye trackers in this study. This increase of noise levels is seen not only in the gaze signal, but also in the P and CR signals of the eye trackers that provide these camera image features. The P and CR signals of the SMI eye trackers correlate strongly during small head movements, but less so or not at all when the head is completely still, indicating that head movements are registered by the P and CR images in the eye camera. By recording with artificial eyes, we can also show that the pupil size artefact has no major role in increasing and colouring noise. Our findings add to and replicate the observation by Niehorster et al., (2021) that lowpass filters in video-based P–CR eye trackers colour the data. Irrespective of source, filters or head movements, coloured noise can be confused for oculomotor drift. We also find that usage of the default head restriction in the EyeLink 1000+, the EyeLink II and the HiSpeed240 result in *noisier* data compared to less head restriction. Researchers investigating data quality in eye trackers should consider not using the Gen 2 artificial eye from SR Research / EyeLink. Data recorded with this artificial eye are much noisier than data recorded with other artificial eyes, on average 2.2–14.5 times worse for the five eye trackers.

## Introduction

When characterising the quality of data produced by eye trackers, precision – the level of variability in the data, independent of its source – is one of the most important properties to take into account (BiPM et al., 2012). Although much progress has been made in characterising precision (e.g. Niehorster et al.,, 2020b; Wang et al.,, 2017; Blignaut and Beelders, 2012; Coey et al.,, 2012), several unresolved questions remain.

Variability in eye-movement data is thought to arise from at least two sources, 1) noise inherent in the measurement device (Niehorster et al., 2020b; Blignaut & Beelders, 2012), and 2) rotations of the eyeball itself, such as tremor, drift, and microsaccades (Ratliff & Riggs, 1950; Collewijn & Kowler, 2008; Martinez-Conde et al., 2004; Rolfs, 2009). In this paper, we want to direct attention to the role of very small head movements on the precision of data from eye trackers.

Modeling eye trackers geometrically and physically is one way to investigate the quality of the tools we are using for our research (Hansen & Ji, 2010). Our approach is to use empirical methods to investigate commercial eye trackers with unknown/unpublished settings, algorithms, sensor properties etc. Because eye trackers are the participants of data-quality studies, we take the approach that we should investigate eye trackers with the same methods as we use when investigating the psychology of human beings.

### Precision - the *level* of noise

That precision has a major effect on reliable detection and classification of events such as microsaccades, but also for detecting drift, fixations and regular saccades has been shown by van Renswoude et al., (2018), Hessels et al., (2017), and Holmqvist (2016), and Holmqvist et al., (2012).

Precision in data from eye trackers is known to be influenced by a number of factors; for instance, human eyes have been found to cause noisier data than do artificial eyes (Niehorster et al., 2020b), different eye trackers produce recordings of different levels of precision (Holmqvist, 2015), and blue eyes result in noisier data than brown eyes (Hessels et al., 2015a; Holmqvist, 2015).

The level (or magnitude) or precision is often measured as RMS-S2S (the root mean square of the sample-to-sample distances), which reflects the high velocity noise that is characterised by large sample-to-sample movements in the gaze signal. Sometimes precision is instead measured as standard deviation (STD), which ignores the size of sample-to-sample movements but instead measures the dispersion of gaze (i.e. its spread over space). (For mathematical definitions, see Niehorster et al.,, 2020b). We will speak of RMS-S2S and STD precision when referring to these two operationalisations.

### Precision - the *colour* of noise

The most common way to calculate colour of noise in a signal is to perform a spectral density (PSD) analysis (Niehorster et al., 2020b; Wang et al., 2017; Coey et al., 2012). It is commonly found that dynamical systems follow power-law scaling behaviour of the signals power with frequency, as described by the formula

1 $$ S(f) \propto \frac{1}{f^{\alpha}} $$

where *S*(*f*) is the PSD of 1D gaze (or some other signal), as a function of signal frequency. The slope *α* is a scaling component that can be used to assess the spectral colour of the gaze signal. For white noise, where the energy at each frequency is the same, *α* is zero. For coloured noise, *α* takes on higher values, because the signal is more energetic at some frequencies (typically lower frequencies).

Although *α* is the standard way to calculate colour, there are alternative methods. Niehorster et al., (2020b) could recently show that the quotient ${\frac {\text {RMS-S2S}}{\text {STD}}}$ maps onto the slope *α* of the power-spectral density plot of the same segment of data. The relationship was established using real and simulated gaze data; see the paper for details. This entails that both *α* and ${\frac {\text {RMS-S2S}}{\text {STD}}}$ can be used to describe the colour of the data. We will follow Niehorster et al., (2020b) in defining a shorter name Type as:

2 $$ Type = \frac{\text{RMS-S2S}}{\text{STD}} $$

Type refers to the shape of the signal in 2D space: spiky vs smooth (see Figure 5 in Niehorster et al., 2020b). Slow noise results in smooth signals with low Type values, while high-velocity noise results in spiky signals with higher Type values. White noise has a Type value of $\sqrt {2}$. Throughout this paper, we will use Type to investigate colour of noise in the gaze signal from eye trackers, instead of *α*.

Interestingly, the colour of the recorded gaze data, the Type, differs slightly between data recorded from human vs artificial eyes (Niehorster et al.,, 2020b, Figure 10): Unfiltered human gaze data have some slight colour, while unfiltered data from artificial eyes are completely white. The authors speculate that human oculomotor drift, the pupil size artefact or head movements can cause this slight colour. One goal of this paper is to show which of these three sources contribute to colour of gaze data.

All our calculations of precision, as RMS-S2S, STD and Type, are made on 2D gaze signals (x,y on the monitor) or 2D camera image features (x,y on the camera sensor).

### Possible explanations to increased imprecision and colour of noise in human eye-tracker data

Several geometrical models of gaze estimation in eye trackers have been published, and they do give us good insights in how small head movements could result in inaccuracies in the reported gaze data (Hansen and Ji, 2010, e.g.). Testing several geometrical models, Cerrolaza et al., (2012) showed that inaccuracies may originate from small stabilizing head movements that participants make. However, as far as we can tell, none of these models predict a poorer *precision* due to very small head movements.

Some level of imprecision likely originates from noise produced by pupil and corneal reflection (CR) center calculation algorithms operating on the image at the camera sensor of the eye tracker. For instance, errors in the center calculation of the CR image of the eye camera sensor was proposed by Holmqvist and Blignaut (2020) as the most likely explanation for substantial mismeasurements of the amplitudes of small saccades. In all tested video-based eye trackers, small eye movements were mismeasured such that, for instance, a 10$^{\prime }$ (arcmin) microsaccade was sometimes reported with an amplitude of 5$^{\prime }$, and sometimes with an amplitude of 25$^{\prime }$. These CR errors repeated over the measurement space with a wavelength that varied between 30–120$^{\prime }$, depending on the eye tracker (Holmqvist & Blignaut, 2020, Table 6). If such errors happen during very small translational or rotational head movements, it should generate small movements in the gaze data signal that could be interpreted either as noise or artificial eye movements. Such a mechanism would explain the higher noise (poorer precision) in data from human eyes compared to data from artificial eyes, as reported by Niehorster et al., (2020b), and possibly also the increased colouring of the data.

Furthermore, it is conceivable that an uneven shape of the human cornea (Kiely et al.,, 1982 e.g) may lead to small random and erroneous movements in the CR and subsequently in the gaze signal, which could manifest themselves as noise of unclear colouring.

Slow head movements will increase the STD variability more than the RMS-S2S variability, and Equation 2 tells us that Type will then decrease, which means that data will be more coloured.

Lowpass-filtering in the software of some eye trackers is the main reason for colour in the data (Niehorster et al., 2021), but even in unfiltered data, there is a remaining difference in colour of the noise between human and artificial eyes that needs clarification.

Wang et al., (2017) argued that the differences in noise colour between human and artificial eyes stem from the existence of drift and small microsaccades in human data, but this is to a large extent contradicted by Niehorster et al., (2021), and also by Coey et al., (2012).

Furthermore, the pupil size artefact is known to affect gaze accuracy (Hooge et al.,, 2016; Drewes et al.,, 2014, e.g.). Niehorster et al., (2020b) speculate that the pupil size artefact might also be responsible for increased noise and colouring, arguing that the calculation of pupil centers in P-CR trackers is affected by variations in pupil dilation, which means that changes in pupil size could be a contributor to increased noise levels. Since changes in pupil diameter are slow, much like head movements, they could also colour data.

### Hypotheses

In this paper, we set out to investigate whether the level and colour of noise in eye trackers to some part originates from small head movements. For this purpose, we will record from artificial and human eyes in three different configurations:

1. **Static:** The artificial eyes are placed on a static glass head at a fixed distance from the eye tracker, both placed on a vibration-free table. We expect this configuration to result in the lowest level of noise, and that this noise is white for unfiltered data, as previously shown by Niehorster et al., (2020b).
2. **AA:** The same two artificial eyes are placed on a human head, covering the human eyes. This allows us to measure the influence of head movements without any interference from small eye movements. We expect these data to have larger and more coloured noise compared to recording the same artificial eyes mounted on a static glass head. Furthermore, these artificial eyes have pupils with fixed diameters, which would exclude the pupil size artefact as an explanation for the increased noise and colour.
3. **HH:** We record human eyes, which we expect to be noisier than artificial eye data. If we would find more coloured data (lower Type) from humans, as compared to data from artificial eyes (case AA) it would suggest that the pupil size artefact or oculomotor drift is responsible.

## Method

### Experimental design

Our main comparison is between the Static, the AA and the HH conditions. However, based on previous findings, we manipulated a number of factors (Table 1) that have been shown to have an effect on data quality: Firstly, we recorded on five eye trackers (Table 2) that were selected because of their particular properties (Section “Eye trackers”). We also recorded with filters on and off on all eye trackers that allowed it, in order to possibly replicate the finding in Niehorster et al., (2020b) that (lowpass-)filters affect the colour of the recorded noise. Both human participants were recorded in a stabilised and an unrestricted condition, in order to make remote eye trackers more comparable to those that come with a built-in head stabilisation as their default.

**Table 1:** The experimental factors

Factor	#levels	Levels
Eye tracker	5	See Table 2
Filter	2/0	On vs. off where possible
Stabilisation	2	Stabilised vs. unrestricted
Participant type	2	Human vs static
Eyes	4	Human, EL 2, Tobii and SMI
Eye combinations	2	AA and HH (see Section “Masks for attaching artificial eyes”)

**Table 2:** The eye trackers

Eye tracker	Interface	Calibration	Sampling frequency (Hz)	Filtering
SMI HiSpeed 240	Head fixed	Polynomial	240	**On** / off
SMI RED250mobile	Remote	Polynomial	250	**On**
SR Research EyeLink II	Head fixed	Polynomial	250	**On** / off
SR Research EyeLink 1000+	Remote	Polynomial	500	**On** / off
Tobii Spectrum	Remote	3D model	600	**Off**

The P-CR method calculates uncalibrated gaze as the difference between the centres of the pupil image and the CR image in each video frame of the eye. Polynomial calibration uses the solution of a higher-order polynomial equation system to map P and CR data to gaze, while 3D modelling of the eye estimates parameters of the shape of the eye ball. Default filtering marked with bold

Two types of participants took part in the study: Human heads and the static glass head. Humans participated to provide small head movements, whereas the glass head provided data from an immobile baseline setting.

In addition to the human eyes, three types of artificial eyes were evaluate for use in the recordings: the SMI artificial eye, the EyeLink artificial eye, Gen 2., and the Tobii artificial eye (Section “The three pairs of artificial eyes”). Any movement in the data from artificial eyes must reflect either head movements and imperfections in the eye tracker.

### Environment

All recordings took place in a windowless room with no exterior walls. The room had a constant illumination of 145 lux, measured at the point where the artificial eyes were mounted. All light came from fluorescent lamps and the stimulus monitor. Air ventilation was turned off and inroom temperature kept stable at 21^∘^ Celcius. The eye trackers and the mounting of artificial eyes were placed on a 300 kg vibration-free table. As soon as the recording started and until it ended, noone was allowed to move in the room. All recordings were made during the Bavarian restrictions and curfews of the COVID-19 pandemic, while making sure that no human or machine activity took place in neighbouring rooms. There are no large roads or manufacturing or construction activity nearby that could introduce vibrations.

### The three pairs of artificial eyes

**Fig. 1:**
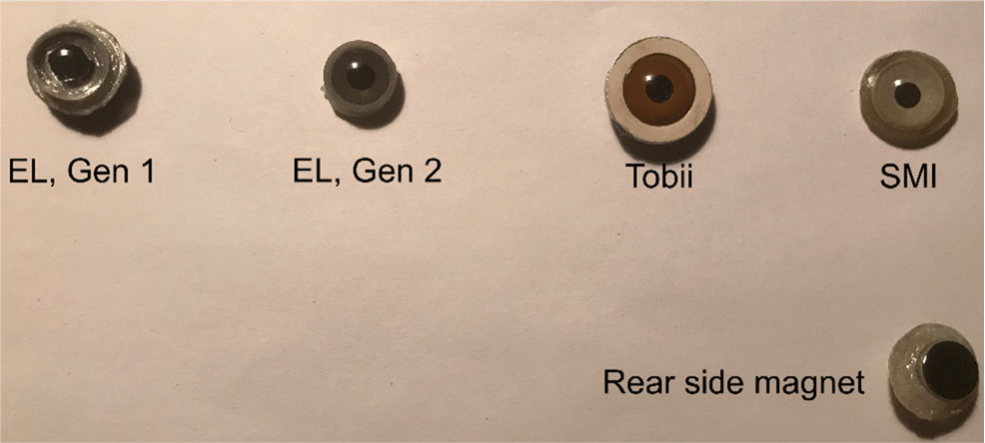
The different artificial eyes. We used EL 2, Tobii and SMI artificial eyes, with a neodym magnet attached on the rear

Tobii AB, SMI GmbH and SR Research have all manufactured their own artificial eyes. This same set of artificial eyes have previously been used by Wang et al., (2017), Niehorster et al., (2020b), and Holmqvist and Blignaut (2020), with the exception that previous studies used the EyeLink eyes of generation 1, while we are using generation 2. Figure 1 shows the artificial eyes in visible light, and the neodym magnets that were glued to the back.

Artificial eyes differ from human eyes in a few aspects. They generally do not have neither a bright pupil reflex nor a lens that can produce a fourth Purkinje reflection. Artificial eyes might be modelled with slightly different corneal shapes and curvatures. Given that human corneas vary in shape (Kiely et al., 1982), it is possible that the even corneal curvature of artificial eyes is more closely align with the model expectations of the five commercial eye trackers we are testing, and that the even cornea will contribute to a lower noise than with human data. Artificial eyes have fixed pupil sizes of around 4 mm rather than a variable pupil dilation 2–8 mm which is typically seen in humans. Artificial eyes are made of a different material than found in human corneas, and they typically lack moisture. As neither of these features of artificial eyes are likely to affect measurements of precision negatively or unequally between Static and AA conditions, we judged that they are unlikely to have impacted the results found in the current study.

**Fig. 2:**
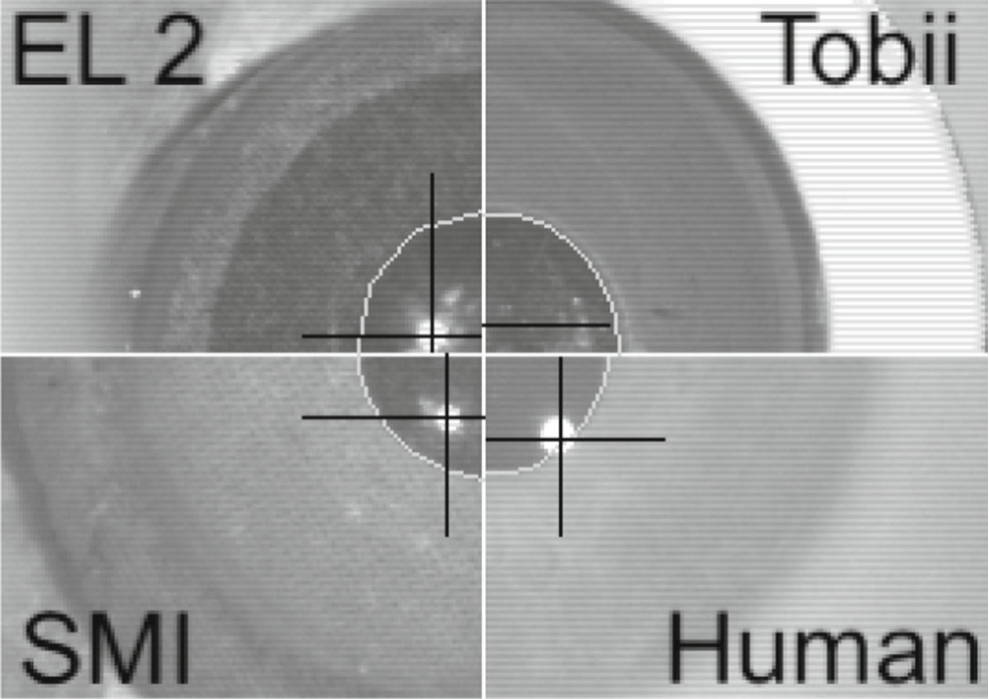
The EL 2, Tobii and SMI artificial eyes, and participant SLÖ as seen in the SMI HiSpeed 240 eye camera

However, the contrast between iris and pupil may very well have an impact on precision levels. As this contrast differs between artificial eyes, we looked at each artificial eyes in infrared light, which is more relevant than looking at them in visible light. Figure 2 shows the three artificial eyes used in the study and human participant SLÖ from the SMI HiSpeed 240 camera. Notice that the contrast between iris and pupil is poorer in the EL 2 eye compared to the other three eyes. Also notice that all three artificial eyes exhibit additional, weaker reflections inside the pupil, which however do not seem to disturb the detection of the actual corneal reflection.

### Heads for generating head movements

Two humans heads, belonging to authors KH and SLÖ, provided human head movements for the HH and AA conditions. The human participants also provided their own human eyes for the HH condition.

KH has 25 years of experience of eye tracking, light blue eyes and used no visual aids. SLÖ has one year of experience of eye tracking, brown eyes and used no visual aids. Both humans have left dominant eyes and upward/forward directed eye lashes. No make-up was used. Both KH and SLÖ exercise regularly, have good muscle control, and can sit very still.

**Fig. 3:**
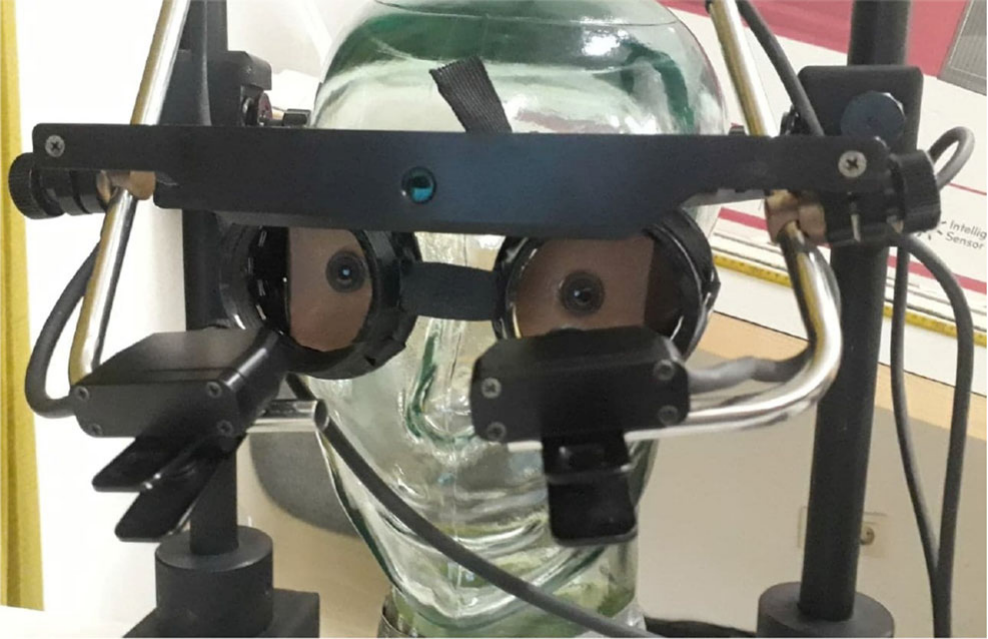
The static glass head, wearing the mask on which the artificial eyes were mounted. The picture also shows the modified version of the EyeLink II, which represents the unrestricted condition for this eye tracker

The static, entirely immobilised glass head was used for recording baseline (Static) data in which no head movements are made (Fig. 3). For the Tobii Spectrum and SMI RED250mobile, we had to attach a face mask to the glass head in order for the trackers to accept the artificial eyes; this very same face mask was previously used by Holmqvist and Blignaut (2020).

### Masks for attaching artificial eyes

Human eyes (HH) were recorded as in any eye-tracking study. Artificial eyes (AA and Static) were mounted on modified protection glasses (Fig. 3). We replaced the glass cover with cardboard to cover the human eye, and glued a metal plate onto the cardboard, which allowed a very stable attachment of the neodym magnets at the rear of the artificial eyes. The protection glasses had a snug fit onto both human (AA) and glass heads (Static). The setup with strong magnets also made it easy to switch artificial eyes between recording trials, and to make small adjustments to the exact position of artificial eyes during setup.

We also covered all metal surfaces with black tape to prevent erroneous reflections, and added a thin paper between the artificial eye and the metal plate, for the same reason.

### Eye trackers

We recorded on the Tobii Spectrum, the SMI HiSpeed 240, the RED250mobile, the SR Research EyeLink 1000+ at 500 Hz, and the EyeLink II. The two EyeLinks were chosen because they have been extensively used not only in microsaccade research, as evidenced by Table 1 in Martinez-Conde et al., (2009), but also for measuring oculomotor drift (Roberts et al., 2013; Engbert and Mergenthaler, 2006). The SMI HiSpeed 240 is a well-examined eye tracker, with very regular measurement artefacts. The Tobii Spectrum is the flagship eye tracker of the largest manufacturer, and the SMI RED250mobile was chosen because it exhibits the largest errors in amplitude measurements (Holmqvist & Blignaut, 2020). That said, these five eye trackers are all P-CR video eye trackers, and Holmqvist & Blignaut found that all five have clear CR artefacts that could turn head movements into noise and artefacts in the gaze signal.

For all eye trackers, we recorded each condition in a stabilised and an unrestricted setup. For the Spectrum and the RED250mobile, we simply recorded with and without a chinrest. For the HiSpeed 240 and the EyeLink 1000, we recorded with the standard chin- and forehead-rest for the stabilised condition, and only with forehead-rest in the unrestricted condition. The EyeLink II was recorded in two human interface configurations. In the first case, we used the ordinary head-mount of the system. In the second case, we dismantled the head-mount and attached it to a modified chinrest mounted on the table, such that the eye tracker is not touching the participant head (Fig. 3). These two cases represent the stabilised and unrestricted conditions of the EyeLink II.

Whenever possible, we recorded all conditions with and without the standard lowpass filters. For the EyeLinks, we used the “no filter” and “extra filter” settings, while for the HiSpeed 240, we turned on/off both the bilateral and the heuristic filters. On the SMI RED250mobile and the Tobii Spectrum, no filter settings are available, but we know from Niehorster et al., (2020b) that the RED250mobile gaze data are filtered, and Tobii claim that the Spectrum gaze data are unfiltered by default (which our analysis below confirms).

Since the Spectrum automatically switches between dark pupil and bright pupil mode and this may disrupt the recording of artificial eyes (which are unable to produce a bright pupil), we covered most of the illuminators around the two eye cameras of the Spectrum, such that we force the eye tracker to record in dark pupil mode. This was done by placing halved, circular paper cut-outs with a hole in the middle, so as not to obstruct the view of the camera, above the outer half of the bright pupil illuminators. This allowed us to record on the Spectrum both humans and artificial eyes in the same dark pupil mode.

Both the Spectrum and the RED250mobile require a face around the artificial eyes to record data. Furthermore, both remote eye trackers recorded more precise data if artificial eye lids in skin colour were placed above and below the artificial eyes. For all other eye trackers, it is enough to place the artificial eyes in front of them, and they start tracking.

### Procedure

We first calibrated each eye tracker on human eyes using the built-in manufacturer software, always with a 9 point calibration. If the recording software allowed us to save and later reuse a calibration, we did so for new recordings of the Static and AA condition. The procedure to calibrate for other eyes than the eyes recorded has been used for recordings with artificial eyes by Holmqvist and Blignaut (2020) and Niehorster et al., (2020b) and Wang et al., (2017), and has been shown by Holmqvist and Blignaut (2020) and Harrar et al., (2018) to produce no non-linearities in data, but slightly poorer accuracy, which we presume has no bearing on precision.

We took care to set up the eye camera and P and CR thresholds on the EyeLinks and the SMI HiSpeed such that centre calculation of each feature was unobstructed and as free of noise as possible.

Data were then recorded while participants watched a single fixation point in the middle of the recording area for the duration of 100 s, while remaining as still as possible.

On the two remotes (Tobii Spectrum and SMI RED250 mobile), we recorded 10 ×10 s, to allow for readjustments every 10 s, if we were seeing deteriorating data quality in online eye images or gaze cursors. This was necessary, because the two remote systems were difficult to record on, as experienced also by Holmqvist and Blignaut (2020). Both when a human was wearing the AA mask as well as when the eyes were mounted on the static glass head, these two eye trackers struggled with recognising the eyes, and easily lost tracking if the setup was not optimal. Setup time for a single recording could be more than half an hour of trial and error. The quality of the recording seemed to depend on a multitude of factors, but was primarily influenced by the exact angle and position of the artificial eyes, the distance from the screen, and the type of artificial eye used.

### Data processing

First we calculated precision in terms of RMS-S2S and STD, using a similar procedure as in Niehorster et al., (2020b). Each approximately 100 s long data recording was split into trials: non-overlapping segments of 2 seconds. Table 3 summarises the number of trials per eye tracker and eye. Data before each recording pause, if any, were discarded if it was not 2 s long. For each of the trials, dispersion was calculated separately for the left and right eye and then combined into one dispersion measure by calculating the l2-norm of the two. The 50% of the trials with the highest dispersion were discarded from further analyses.

**Table 3:** Number of trials, i.e. non-overlapping 2 second segments of data, per eye tracker and eye

Eye tracker	Eye	Static	AA	HH
HS240	H	–	–	232
HS240	S	51	221	–
RED250	H	–	–	98
RED250	S	25	69	–
EL II	H	–	–	208
EL II	S	50	200	–
EL1000	H	–	–	213
EL1000	S	52	200	–
Spectrum	H	–	–	99
Spectrum	T	24	98	–

There are less data for the Spectrum and RED250 because they have only one filtering option

RMS-S2S and STD were calculated using the remaining trials and a sliding window of 200 ms with a 50 ms stride. To avoid potential microsaccades, only windows with dispersion below the median were included in RMS-S2S and STD analyses. The remaining number of 200 ms windows are listed in Table 4.

**Table 4:** Number of windows, i.e. 200 ms windows from which our data points are calculated, per eye tracker and eye

Eye tracker	Eye	Static	AA	HH
HS240	H	-	-	4290
HS240	S	938	4087	-
RED250	H	-	-	1862
RED250	S	475	1306	-
EL II	H	-	-	3952
EL II	S	949	3794	-
EL1000	H	-	-	3933
EL1000	S	962	3684	-
Spectrum	H	-	-	1687
Spectrum	T	441	1794	-

There are less data for the Spectrum and RED250 because they have only one filtering option

Finally, we recalculated all RMS-S2S and STD values to degrees for each separate eye tracker, using the specific geometry of the recording (monitor size, resolution and participant-monitor distance), and divided RMS-S2S by STD to form Type (${\frac {\text {RMS-S2S}}{\text {STD}}}$). All comparisons of RMS-S2S, STD, and Type of gaze, P and CR are made within eye trackers (i.e. within-subjects) and between conditions Static, AA and HH.

All plots and statistics were made in R, version 3.6.1 (Core Team, 2013). We present all results pertaining to gaze as angular error. Results for P and CR are reported in pixel coordinates of the camera sensor. Remaining figures are reported in the unitless Type, quantifying the colour of noise.

## Results 1: Selection of artificial eyes to use in subsequent analyses

**Fig. 4:**
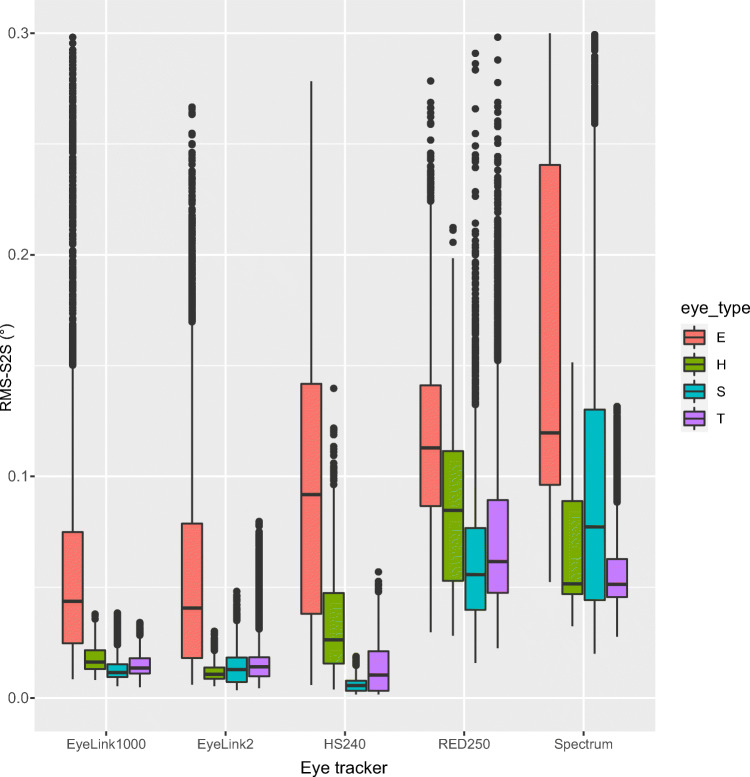
RMS-S2S precision of 2D gaze, for the five eye trackers, and the four types of eyes. Note how much worse precision the EyeLink Gen 2 eyes (E) produce compared to Human (H), SMI (S) and Tobii eyes (T). Default filtering was applied to all trackers in this plot (on for all but the Tobii Spectrum), and data from both stabilized and unstabilized recordings were included

Figure 4 plots the RMS-S2S distributions of each artificial eye and each eye tracker. For all eye trackers, except the Tobii Spectrum, the SMI eyes resulted in the lowest (best) precision, and the narrowest distributions. In particular, the EyeLink 2 artificial eyes produced much noisier data compared to other eyes on all eye trackers, and much wider distributions. Average RMS-S2S was 2.2–14.5 times worse for the EyeLink 2 eye compared to the eye with the lowest RMS-S2S. The most likely reason for the poor RMS-S2S precision and the wide distributions is the poor contrast between the pupil and the iris of the EyeLink 2 artificial eye (see Fig. 2). Such a poor contrast between iris and pupil would only be found for rare human eyes having a dark blue eye colour (Figure 4.13 in Holmqvist and Andersson, 2017).

In all subsequent analyses, we will therefore use data from the SMI artificial eyes, except when using data from the Tobii Spectrum where we employ data recorded with the Tobii eyes. We will not employ the EyeLink 2 artificial eye in any of the analyses.

## Results 2: Level and colour of noise in the gaze signal

### The effect of built-in filters on the colour of noise

**Fig. 5:**
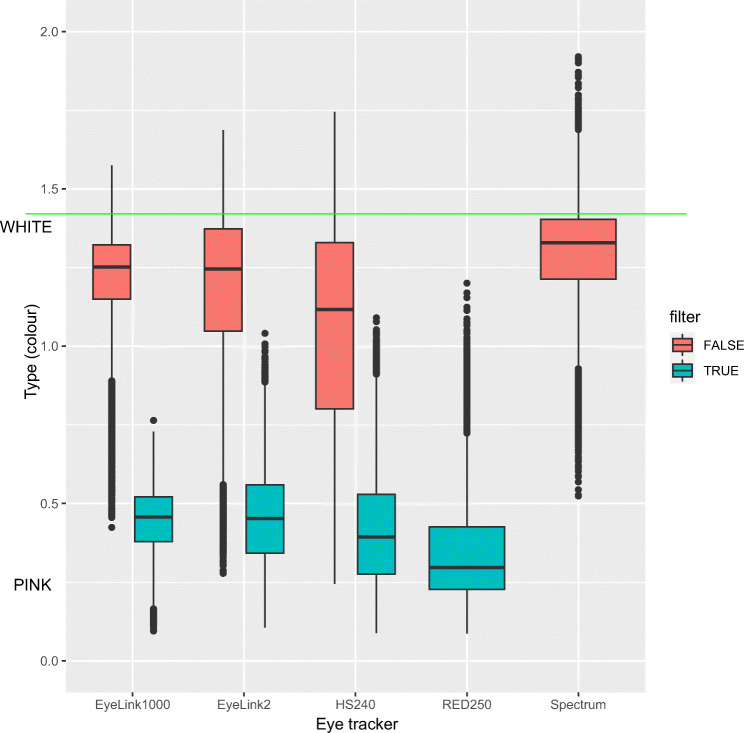
Type precision of 2D gaze, for the five eye trackers, with and without filtering. Data from both stabilized and unstabilized recordings were included. White noise corresponds to Type values near the square root of 2 (indicated with a green line), while coloured noise has values nearer to 0

Figure 5 compares the colour of noise using the Type measure. Results show that filters colour data (lower Type), which replicates the finding in Niehorster et al., (2020b, Fig. 10). The result in Fig. 5 is also compatible with Coey et al., (2012) and Blignaut and Beelders (2012), but it is inconsistent with the findings reported by Wang et al., (2017). Also notice how the SMI RED250mobile is coloured (filtered) by default, while the Spectrum has white (unfiltered) data by default. Neither the Tobii Spectrum, nor the SMI RED250mobile allow the user to change the filtering of data.

### The effect of small head movements on the level and the colour of noise

**Fig. 6:**
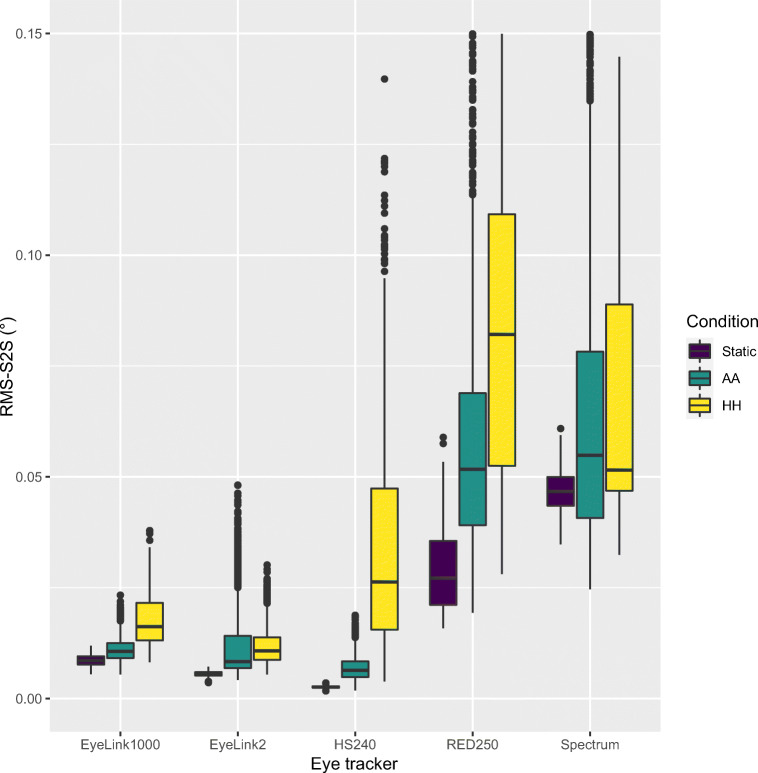
RMS-S2S precision of 2D gaze, for Static glass head with artificial eyes, artificial eyes on human head (AA) and human eyes in a human head (HH). The SMI artificial eyes were used for all eye trackers, except the Tobii Spectrum, where we used the Tobii artificial eyes. Default filtering was used for all eye trackers

We then compared 2D RMS-S2S, STD and Type between the Static glass head with artificial eyes (Static), artificial eyes on human head (AA) and human eyes in a human head (HH). Figure 6 shows how RMS-S2S precision is lowest (best) for the Static glass head with the optimal artificial eye, while the second lowest (best) precision is for the same artificial eyes worn on a human head, and the noisiest data for all eye trackers is with human eyes, with the exception for the Tobii Spectrum.

The finding in Fig. 6 has the interesting implication that head movements do contribute to the increased noise levels in all the five eye trackers. Because data from human eyes (HH) are even noisier than data from artificial eyes worn by humans (AA) for four of the eye trackers, there is a possibility that pupil artefacts or fixational eye movements, besides head movements, also contribute to the higher noise levels. It is unclear why the Tobii Spectrum is different, but it is likely that their tracking of head movements works better, or that the difficulty to record on the Spectrum resulted in a lack of the expected effect due to increased measurement noise.

Niehorster et al., (2020b, Fig. 10) show a difference in the colour of noise between artificial and human eyes, with human eyes producing slightly more coloured noise than the artificial eyes, also for unfiltered data. If this difference in colour would depend only on the type of eye (human or artificial) and not on the type of head (human or static), the colour of noise in data from artificial eyes recorded on the static head should be the same as from artificial eyes recorded on a human head (AA).

**Fig. 7:**
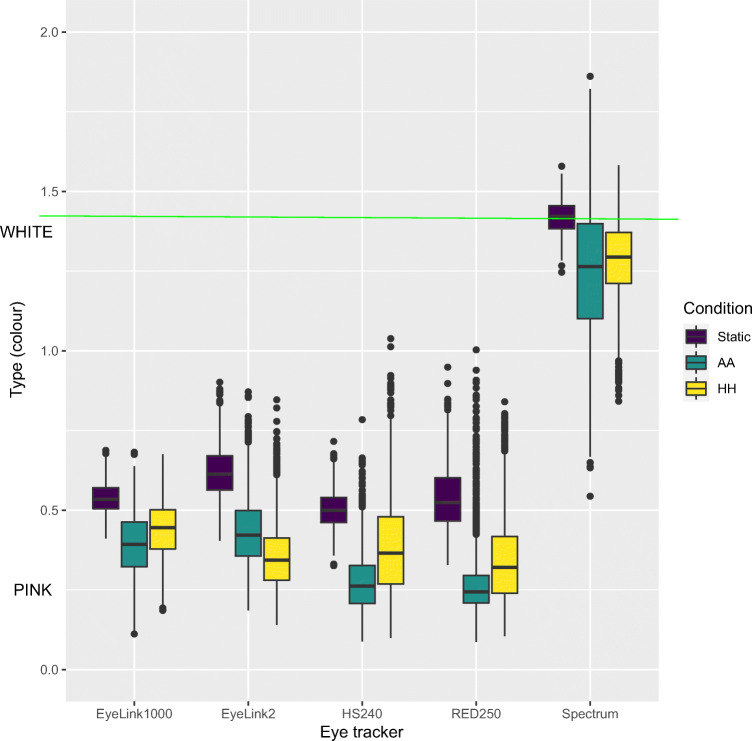
Type of 2D gaze, for Static glass head with artificial eyes, artificial eyes on human head (AA) and human eyes in a human head (HH). The SMI artificial eyes were used for all eye trackers, except the Tobii Spectrum, where we used the Tobii artificial eyes. Default filtering was used for all eye trackers

However, Fig. 7 shows that gaze data recorded from artificial eyes on a human head (AA) are more similar in colour to data recorded from human eyes (HH) than they are to data from artificial eyes recorded on a static head (Static). The finding in Fig. 7 provides support for the conclusion that head movements play the major part in creating the differences in noise colour between human and artificial eyes, and that the pupil size artefact speculated by Niehorster et al., (2021) to be another possible cause, plays no or a much smaller role. Furthermore, the explanation from Wang et al., (2017) that intra-fixational eye movements would colour any of the noise is also not supported by our data, as artificial eyes produce neither microsaccades nor oculomotor drift.

**Fig. 8:**
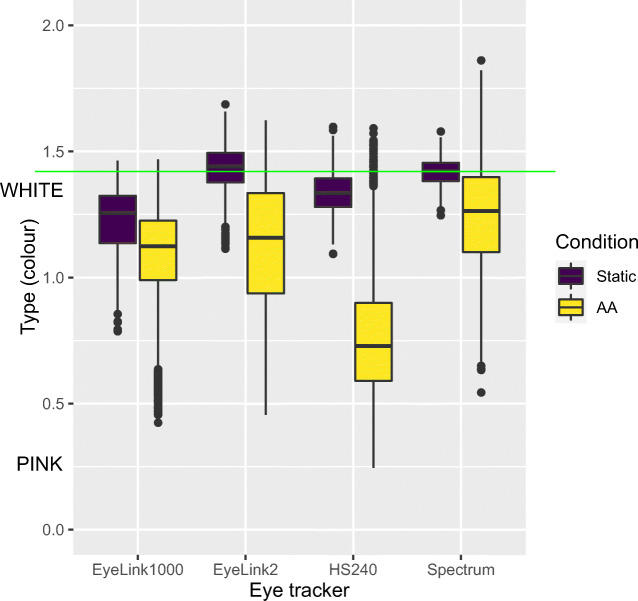
Type of 2D gaze, for Static glass head with artificial eyes, and artificial eyes on human head (AA) for unfiltered data (with and without head stabilization)

In Fig. 8, we show that the same result holds also for unfiltered data: Head movements colour the noise for all eye trackers; more so for some than for others.

**Fig. 9:**
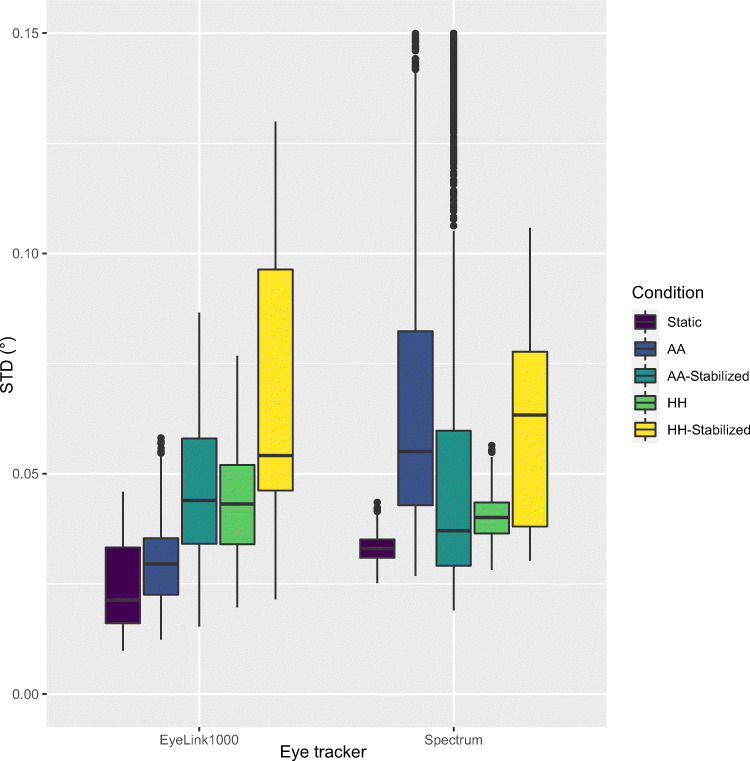
STD precision of 2D gaze, for the EyeLink1000+ and the Tobii Spectrum, comparing stabilized to non-stabilized participants. The EyeLink II and the HiSpeed240 exhibit a very similar effect to the EyeLink 1000, while the SMI RED250mobile has the same unclear effect of stabilization as the Tobii Spectrum

How can head movements change the colour of noise? The colour of noise, Type (${\frac {\text {RMS-S2S}}{\text {STD}}}$) decreases if RMS-S2S increases less than STD. RMS-S2S is a measure of velocity noise in the gaze signal, while STD is a measure of dispersion. Small and slow head movements will *increase STD more than RMS-S2S*. Indeed, the data for STD precision (Fig. 9) shows the exact same trends as the RMS-S2S precision in Fig. 6 but with even larger effect sizes. Hence, Type (${\frac {\text {RMS-S2S}}{\text {STD}}}$) decreases, and we find the result in Fig. 7.

### The effect of head stabilisation on noise

The results in the previous section are based on data in which half the trials involve free seating and the other half were recorded using a full chin- and headrest. It is conceivable that head movements can be sufficiently constrained by a combined chin- and headrest (as speculated by Niehorster et al.,, 2021), which would potentially affect our results. We therefore compare data recorded with versus without restriction on all five eye trackers.

Figure 9 shows the STD precision with and without restriction for two representative eye trackers. The EyeLink1000+ exhibits *noisier* data with combined chin- and forehead rest (stabilized) than with just a forehead rest, while the Spectrum data show mixed results. The EyeLink II and the HiSpeed240 exhibit a very similar effect to the EyeLink 1000+, which is not surprising as they are similar in their construction. In contrast, the RED250mobile is a remote with unknown head movement compensation algorithms, and exhibits the same unclear mix of results as the Spectrum.

## Results 3: Positions of P and CR images on the eye camera sensor

Head movements should directly affect the position of the P and CR images in the eye camera sensor of the eye tracker. We do not know the algorithms that calculate P and CR centers from the eye images in commercial eye trackers, but we do know that open-source algorithms for calculating the centre points often differ between P and CR images (Hosp et al.,, 2020; Mestre et al.,, 2018, e.g).

In this third result section, we investigate the behaviour of the P and CR signals from the two eye trackers that provide them. This is important because gaze, which we investigated in the first two result sections is calculated from P and CR, in some cases simply as P minus CR.

### The effect of small head movements on the processing of P and CR signals from the eye camera

**Fig. 10:**
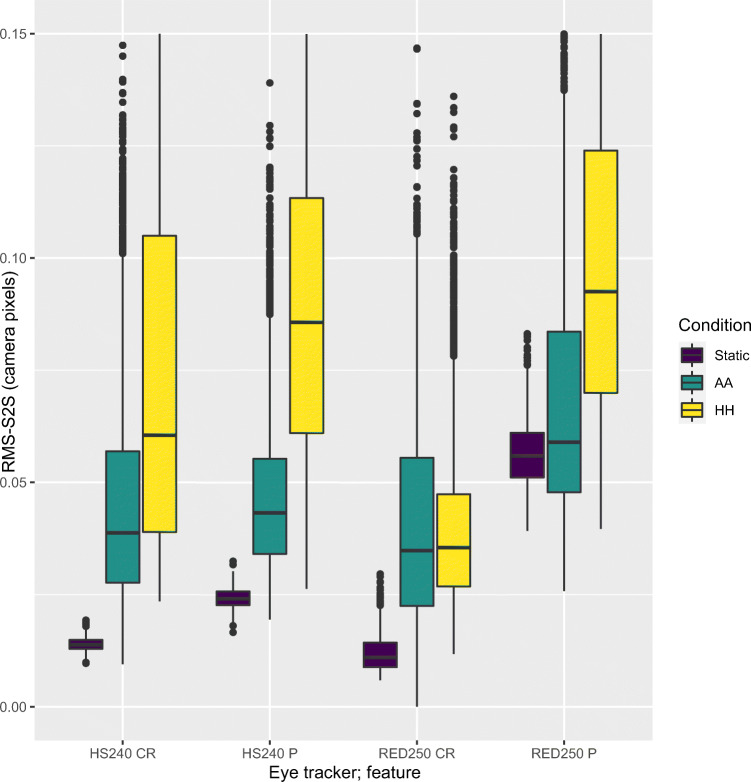
RMS-S2S of the 2D Pupil center signal (P) and the CR center signal from the processing of the eye camera images. Only SMI eyes were used for static and AA conditions. The unit of RMS-S2S in this figure is camera pixels; with most values in the range 0.02–0.10 px

In Fig. 10 we plot the RMS-S2S noise of the P and CR signals for the HiSpeed 240 and the RED250mobile (the other eye trackers did not supply these data). Figure 10 shows that as soon as the artificial eyes are mounted on a human head, we find elevated (worse) RMS-S2S precision of both the P and CR signals: head-movements cause increased noise in the AA condition for both P and CR signals, compared to the Static condition.

Furthermore, as Fig. 10 shows, the RMS-S2S noise is mostly larger in P than in CR for both systems and most conditions, replicating the finding for the HiSpeed 1250 in Table 1 of Hooge et al., (2016). Since camera noise is the same for each image, this result implies that the algorithms indeed differ in how the P and CR images are processed into signals.

### Do the P and CR signals move similarly and synchronously?

In order to examine whether there is movement of related sizes in the noise of the two signals, we correlated the P and CR signals, both as range and as STD, for all 200 ms measurements windows in each condition, because these measures reflect slow changes in the signal. For this analysis, in order to avoid outliers potentially affecting the correlation score, we first applied a RANSAC (RANdom SAmple Consensus) linear regressor^1^, and removed detected outliers prior to calculating the Spearman correlations.

Table 5 shows that for both eye trackers, in both the AA and the HH conditions, the size of the movements of the CR centre and the pupil centre are indeed strongly correlated: When a small head movement takes the artificial eye one way, both P and CR signals follow suit. These movements are a tiny fraction of a camera pixel, according to Fig. 10. However, in the Static condition we find minimal or no correlations between the movement of P and CR signals, for both eye trackers. The amplitude of these Static movements are even smaller than for AA and HH movements, most likely resulting from noise in the camera image and from algorithms operating on the image.

**Table 5:** Spearman correlations between the P and CR range and P and CR STD for all measurement windows per condition, including both filtered and unfiltered data (which does not affect P and CR)

ET	Condition	Restriction	Range	STD
RED	Static		0.363	0.382
250	AA	Yes	0.718	0.765
	AA	No	0.993	0.996
	HH	Yes	0.750	0.765
	HH	No	0.990	0.993
HS	Static		-0.038	0.087
240	AA	Yes	0.975	0.990
	AA	No	0.968	0.979
	HH	Yes	0.860	0.921
	HH	No	0.923	0.949

Data from the RED250mobile and the HS240 are used here, recorded with the SMI artificial eye. All correlations are significant with p-values below 10^− 31^, except in the static condition of the HS240, which is not significant

**Fig. 11:**
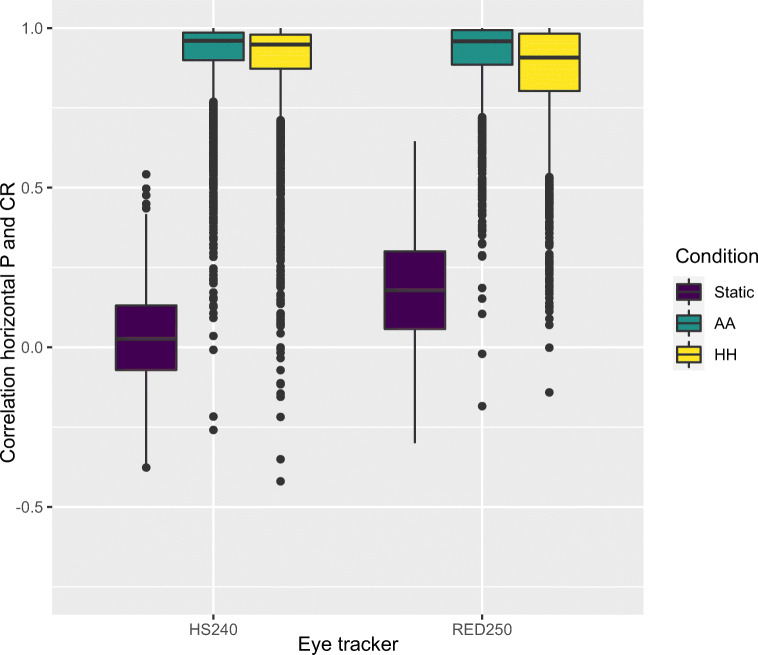
Boxplots of Spearman correlations between horizontal P and CR signals in each analysis window. Only SMI eyes were used for static and AA conditions

**Fig. 12:**
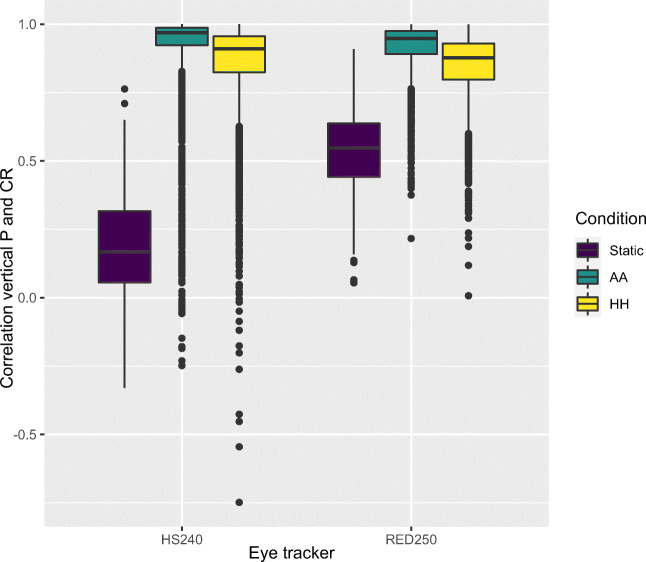
Boxplots of Spearman correlations between vertical P and CR signals in each analysis window. Only SMI eyes were used for static and AA conditions

In order to examine whether these movements are also similarly directed, we correlated the raw P and CR signals of the HS240 eye tracker, for each 200 ms window, using Spearman correlations. Figure 11 shows the correlations between the horizontal P and CR components while Fig. 12 shows the corresponding correlations of the vertical P and CR. For both the RED250mobile and the HS240, the head-worn eyes of AA and HH produce strong correlations between P and CR, while for the Static conditions, there is no correlation for HS240, and moderate ones for the RED250mobile. This shows that head movements produce synchronous and similarly directed changes in P and CR signals, representing movement of those images over the camera sensor. The slightly lower correlations for the HH condition compared to AA suggests that eye movements may indeed reduce the similarity in movement between P and CR, exactly what models of P-CR tracking would predict (Hansen and Ji, 2010). When the correlation between P and CR is below 1, P and CR centers move differently on the eye camera sensor, and the gaze signal will show either high-velocity noise or an eye-movement. Both Figs. 11 and 12 show that for some analysis windows, this happens, in both AA and HH condition; as seen by the many values between 1 and 0, and even negative correlations in the lower end of the distributions.

### The effect of mostly translational movements of artificial eyes on the P and CR signals

**Fig. 13:**
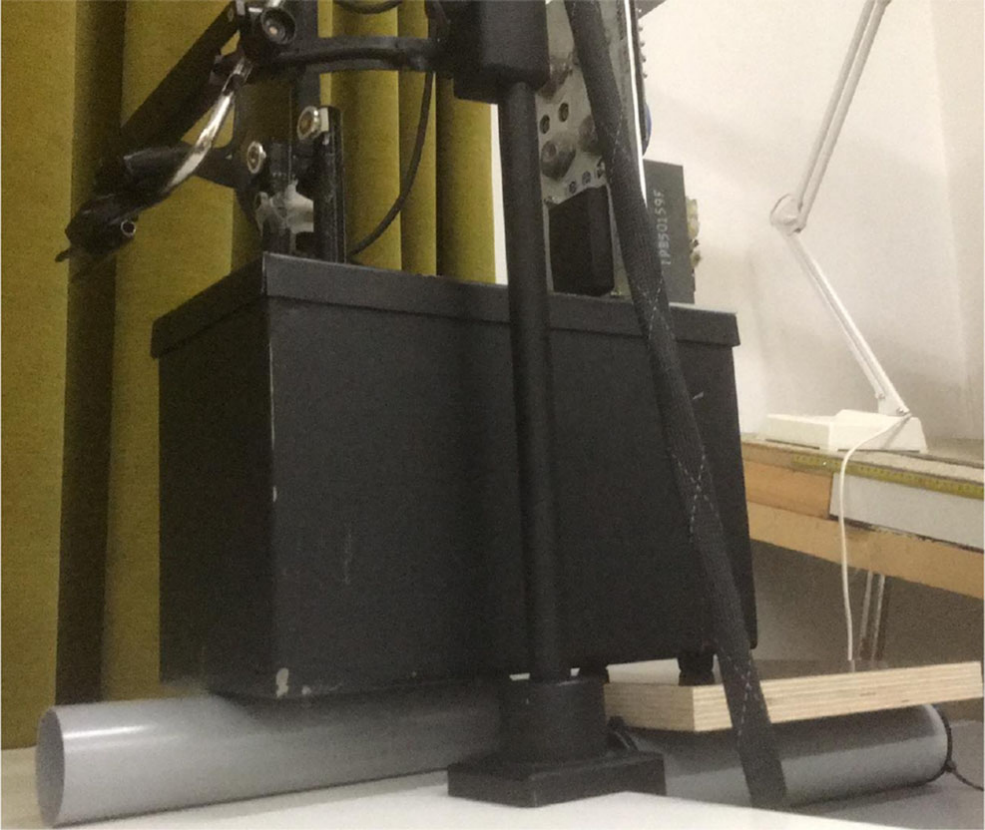
The Stepperbox in a setup that allows for translational movement of a pair of artificial eyes

All previous head movements (AA and HH) have been very small and slow. In order to test whether the P and CR signals are misaligned during larger head movements, of up to 2 centimeters, we built a simple mechanism to move artificial eyes sideways, with minimal rotations. We attached the artificial eyes onto the Stepperbox from Holmqvist and Blignaut (2020), which provided a heavy and stable foundation for letting the artificial eyes stare straight ahead. The Stepperbox and an additional weight were placed onto a small board that smoothly rolled on two plastic tubes (with an extra tube for the Stepperbox). Springs on the tubes ensured a soft halt and that the endpoints of the translation keep the artificial eyes within the eye video frame of each eye tracker. We took care that the edges of all three tubes moved alongside the edge of the table where the eye tracker was mounted (Fig. 13).

We then recorded artificial eye data for the EyeLink II and the HiSpeed 240 over 30 seconds of back and forth, manually induced translational movements of approximately two centimeters, to simulate mostly translational head movements. For the recorded SMI HiSpeed 240 data, we modelled the CR signal based on the pupil signal, using a linear regression from pupil to CR. Table 6 shows the slope is around 1.2. We also performed the same linear regression modelling for rotational data from Holmqvist & Blignaut (1-10 and 10-100 arcmin rotations), and found radically different slopes (about -0.3) and intercepts (Table 6). This suggests that the basic difference between translation and rotation can be readily distinguished by the SMI HiSpeed 240, at least for translations of a few centimeters and rotations of a few minarcs and above.

**Table 6:** Modeling the Pupil movement based on the CR movement, in camera pixels, using a simple linear regression

Movement	Eye	Slope	Intercept	R^2^
Translation	SMI	1.188	0.661	0.998
Rotation 1	SMI	-0.337	125.89	0.318
Rotation 2	SMI	-0.271	122.02	0.230

All data are recorded on the HiSpeed 240. The rotation data are from Holmqvist and Blignaut (2020), where an artificial eye was rotated

**Fig. 14:**
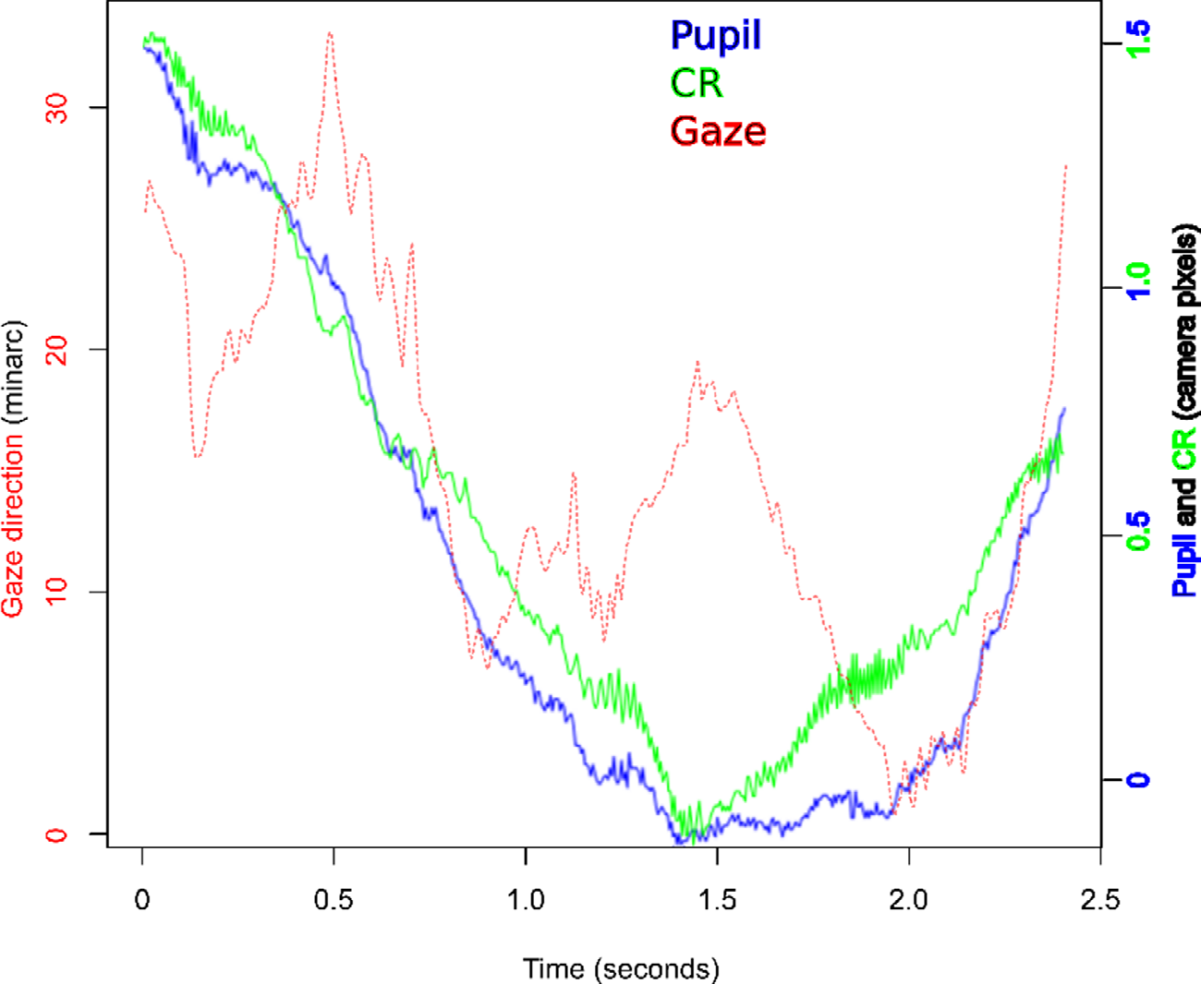
Horizontal CR, Pupil and Gaze signals from the SMI HiSpeed 240 for a typical 2.5 s translational 2 centimeter movement across the camera (appr. 1.5 px movement of CR and Pupil), and the beginning of the return. During these 2.5 s Gaze moves back and forth, of around half a degree, because Pupil and CR signals behave quite differently despite being recorded from the same artificial eye. For optimal illustration, the CR and Pupil signals have been scaled to fit the size of the Gaze plot

Although the HiSpeed 240 appears to be able to largely distinguish between translation and rotation, we do not know how accurately the eye tracker manages to calculate its Pupil and CR signals during mostly translational movements. In Fig. 14, we plot P, CR and Gaze for one and a half translational movement. Note the the P and CR signals should be nearly identical for this largely translational movement, but they are not. This is most likely due to the same artefact found by Holmqvist and Blignaut (2020), that the center calculations for the CR and Pupil images are imperfect, and the variable distances between P and CR cause fluctuations in the Gaze signal that look like irregular rotations of the eye.

The EyeLink II was shown by Holmqvist & Blignaut to exhibit similar artefacts to the HiSpeed 240, but the EyeLink II unfortunately does not output separate Pupil and CR signals. However, we can examine the generation of artificial saccades during translation by looking at the output of the EyeLink saccade detector (which combines a velocity with an acceleration threshold). Table 7 shows that the saccade rate for largely translational movements is between 1.5 to 2.5 saccades per second, and amplitudes are around 1^∘^. Previously, Hermens (2015) showed, using the EyeLink II, that artificial saccades can appear in data as a result of small head movements, which is very much in line with our finding.

**Table 7:** Rate (*s*^− 1^) and average amplitude (^∘^) of artificial saccades for translational movements of the SMI artificial eyes. Data recorded on the EyeLink 2, and saccades detected with the SR event detector

Rate L	Rate R	Ampl L	Ampl R
2.55	1.51	0.89^∘^	1.11^∘^

## Discussion and outlook

We set out to investigate the effect of small, almost imperceptible balance and breathing movements of the body and head on the noise in data from video-based P-CR trackers.

### Head movements affect noise characteristics

In our main comparison, we recorded with completely static artificial eyes, and with the same artificial eyes worn by humans (AA). Comparing the noise from these recordings, we could show that head movements play a clear role to both increased levels (Fig. 6) and an increased colouring of noise seen as lower Type values (Fig. 7). Head movements affect the level of noise not only in the gaze data, but also in the P and CR signals in the two eye trackers that provide those data (Fig. 10). Furthermore, the effect of head movements on noise is the same for filtered (Fig. 6) and unfiltered (Fig. 8) data.

The difference in noise colour in Fig. 7 between static artificial eyes and artificial eyes worn by a human (AA) precludes the pupil size artefact from being the major cause, because the pupil of an artificial eye does not change size. Pupil dilation may nevertheless play a small additional role, because human data are even noisier (but not more coloured).

Because the AA condition involved recordings from artificial eyes which produce neither microsaccades nor oculomotor drift, the slight difference in colour between the AA and the Static conditions (Fig. 6) cannot result from intrafixational eye movements, which the oculomotor hypothesis by Wang et al., (2017) proposes (see also Niehorster et al.,, 2021).

We could replicate the finding in Niehorster et al., (2021) that built-in lowpass filters are a major determiner of the colour of eye movement data. We can therefore repeat and sharpen the observation that researchers who measure oculomotor drift with video-based P-CR eye trackers should be aware that parts of the signal that bear a resemblance to drift may often originate from a combination of filters and head movements.

Niehorster et al., (2021) speculate that it is unlikely that head movements could cause the increased colour in noise, because their participants were restricted by chin and forehead rests. Surprisingly, adding more head support increased the level of noise in the EyeLink1000+, the Eyelink II and the HiSpeed240, but not the remote trackers Tobii Spectrum and the RED250mobile (Fig. 9). This implies that it is disadvantageous to use head restriction in the EyeLink 1000+, the EyeLink II and the HiSpeed240, while it is safe to use head restriction for the SMI RED250mobile and the Tobii Spectrum. One possibility is that the head movement compensation mechanisms of the two remote eye trackers make chinrest usage unnecessary (rather than a problem). It is hard to explain why head restriction leads to noisier data for all eye trackers except the two remotes. One possibility is that sitting in a more restricted setup could lead to more muscle tension and hence more small head movements, or possibly the head tracking algorithms being confused by the headrest. Another possible explanation might be that the data quality of the two remotes was impaired by the difficulty to set them up for tracking with artificial eyes.

### Artificial eyes

Artificial eyes have primarily been used to quantify system noise, that is the precision of the instrument itself, with no influence from human eyes. Figure 4 shows that the RMS-S2S noise in the EyeLink Gen 2 artificial eyes is 2.2–14.5 times worse than in the other eyes and with much larger ranges. We therefore excluded all data from the EyeLink Gen II eyes from our subsequent analyses, and recommend to all eye-movement researchers that they do not use this artificial eye to measure precision.

As we mentioned in Section “4 4”, artificial eyes and human eyes do differ in many ways. Our results to some extent confirm this. Not only is the Gen 2 artificial eye from SR Research / EyeLink unsuited for precision measurements, but more subtle difference exist. In Fig. 6, for instance, we show that RMS-S2S for the HH and the AA conditions differ, which means that the human eyes add some part to noise that the artificial eyes do not, possibly because of varying corneal shapes in combination with the geometrical model. The same pattern is found later, when we look at the RMS-S2S values of the P and CR signals from both human and artificial eyes. However, in other cases the artificial and human eyes have yielded very similar results. For instance, the correlations between P- and CR ranges and STD values in Table 5 show very small differences between human and artificial eyes, suggesting that the movement (dispersion of data points) of the two image centres in the camera sensor happen in the same way for artificial and human eyes. Artificial eyes cannot entirely replace human eyes in data quality research, but for teasing apart the effects of head movements from those of eye movements, artificial eyes are necessary. The same is true for separating system noise from oculomotor noise (Niehorster et al., 2021), investigating how correctly eye rotations are tracked (Holmqvist and Blignaut, 2020), and when measuring end-to-end latencies (Reingold, 2014).

### The P and CR images on the camera sensor

The gaze signal from P-CR eye trackers is formed from the P and the CR signals. We saw in our correlation analyses that P and CR signals very clearly reflect head movements, both in the AA and the HH conditions. In contrast, the P and CR signals appear to move randomly in the Static condition.

We saw in Fig. 10 that both P and CR signals are noisier when recorded in the AA condition (artificial eyes on a human head), compared to the static artificial eyes. We could also see that noise in P is larger than noise in CR, for all conditions and both eye trackers that provide these signals.

Furthermore, Fig. 14 exemplifies the imperfection in the algorithms calculating the centre of the CR and the P signals from the images in the eye camera, the same origin that cause mismeasured amplitudes of small saccades (Holmqvist & Blignaut, 2020). This is most likely where noise and artefacts are produced when the participants head moves ever so slightly.

Hermens (2015) showed that in Pupil-only mode, the EyeLink II produced artificial microsaccades when recording with artificial eyes worn by human participants, and attributed these artefactual microsaccades to the head movement compensation mechanism in the EyeLink II. Recording in P-CR mode decreased the number of generated artificial micro-saccades, but it did not ensure artefact-free recordings. Only when the artificial eyes were mounted on an artificial head did the EyeLink II provided data free of micro-saccades. We could show that when moving artificial eyes sideways across the eye cameras of the EyeLink II, emulating translational body movements in a controlled way, many artefactual small saccades were produced.

All our results pertain to small movements of eyes and head. For larger eye movements, the linear modeling (Table 6) suggests that the SMI eye trackers manages the basic requirement for any P-CR eye tracker to use the P and CR signal to distinguish between translation and rotation. For very large head movements, other artefacts appear (Niehorster et al., 2018).

### Outlook

Our results question the assumption that the eye tracker will record optimal data if participants sit still during recordings. Imperceptible head movements are enough to more than double the noise, colour the signal and generate artefactual eye movements. Seeing this result in the context of other data quality research (e.g. Holmqvist, 2015; Andersson et al.,, 2017; Hooge et al.,, 2016; Wang et al.,, 2019; Hessels et al.,, 2015b; Ehinger et al.,, 2019; Niehorster et al.,, 2020a; Drewes et al.,, 2014; Funke et al.,, 2016), it is clear that researchers who use eye trackers must take care not to make faulty assumptions about their tools, and for manufacturers to think about how to produce better eye trackers.

## Footnotes

1 We used https://scikit-learn.org/stable/modules/generated/sklearn.linear_model.RANSACRegressor.htmlRANSACRegressor function from scikit-learn (version 0.24.0) library (Pedregosa et al., 2011), with X being range or STD of P signal and y – range or STD of CR. We used squared loss and all other parameters as defaults.
